# Aromatic side-chain flips orchestrate the conformational sampling of functional loops in human histone deacetylase 8†

**DOI:** 10.1039/d1sc01929e

**Published:** 2021-05-27

**Authors:** Vaibhav Kumar Shukla, Lucas Siemons, Francesco L. Gervasio, D. Flemming Hansen

**Affiliations:** Department of Structural and Molecular Biology, Division of Biosciences, University College London London WC1E 6BT UK d.hansen@ucl.ac.uk; Department of Chemistry, University College London London WC1E 6BT UK; Pharmaceutical Sciences, University of Geneva Geneva CH-1211 Switzerland; Institute of Pharmaceutical Sciences of Western Switzerland, University of Geneva Geneva CH-1211 Switzerland

## Abstract

Human histone deacetylase 8 (HDAC8) is a key hydrolase in gene regulation and an important drug-target. High-resolution structures of HDAC8 in complex with substrates or inhibitors are available, which have provided insights into the bound state of HDAC8 and its function. Here, using long all-atom unbiased molecular dynamics simulations and Markov state modelling, we show a strong correlation between the conformation of aromatic side chains near the active site and opening and closing of the surrounding functional loops of HDAC8. We also investigated two mutants known to allosterically downregulate the enzymatic activity of HDAC8. Based on experimental data, we hypothesise that I19S-HDAC8 is unable to release the product, whereas both product release and substrate binding are impaired in the S39E-HDAC8 mutant. The presented results deliver detailed insights into the functional dynamics of HDAC8 and provide a mechanism for the substantial downregulation caused by allosteric mutations, including a disease causing one.

## Abstract 2

Mechanistic description of how the active site of a hydrolase is connected with the functional loops at the periphery.

## Graphical abstract

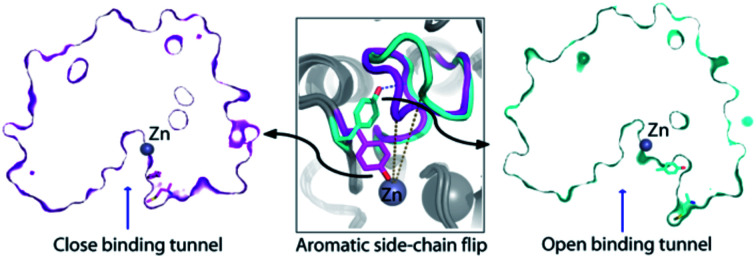

## Introduction

Acetylation of lysine side chains occurs as a co-translation or post-translational modification of proteins and was first identified in histone proteins as a dynamic and reversible process.^1^ The acetylation and de-acetylation reactions are catalysed by histone acetyl-transferases (HATs) and histone deacetylase (HDAC) enzymes, respectively.^1^ Acetylation of histone lysine side chains is often associated with transcriptional activation, whereas de-acetylation often leads to closed chromatin structures and repression of transcription of the underlying genes.^2^ It is therefore not surprising that aberrant acetylation or deacetylation patterns have been linked to several diseases including leukaemia, lymphomas, and neurodegenerative disorders.^3–8^ Substantial efforts are now made to target HDACs for the treatment of such diseases with some HDAC inhibitors already approved by the FDA and several in preclinical trails.

HDACs are traditionally divided into four classes based on sequence similarities. Class I (HDAC-1, -2, -3, and HDAC8), class II (HDAC-4, -5, -6, -7, -9, and HDAC10), and class III (SIRT1-7) have sequence similarity to yeast Rpd3, Hda1, and Sir2, respectively, whereas class IV (HDAC11) shares sequence similarity with both class I and II proteins.^2^ In cancers, class I HDACs act as major epigenetic players and are linked to deregulated expression or interactions with transcription factors critical to tumorigenesis.^9^ Histone deacetylase 8, a class I HDAC, is also implicated in other diseases, including X-linked intellectual disability and parasitic infections.^3,10,11^ Additionally, genetic mutations leading to impaired HDAC8 activity are reported in patients with Cornelia de Lange syndrome (CdLS).^7,8,12–16^

HDAC8 is a unique class I HDAC in terms of its structure, activity, and regulation. Unlike the other three members of class I, HDAC8 is constitutively active, whereas HDAC1 and HDAC2 show activity that increases dramatically once present in multi-protein complexes, and HDAC3 is inactive in isolation.^2^ Moreover, HDAC1, HDAC2, and HDAC3 have serine phosphorylation sites in their flexible C-terminal regulatory tail, which upon phosphorylation activates the enzyme. In contrast, HDAC8 has a regulatory serine phosphorylation site at position 39, in the catalytic domain, and its phosphorylation inactivates the enzyme by stabilising an inactive state.^2,17^ Recently, in HDAC1, HDAC2, and HDAC3, the region corresponding to the S39 site in HDAC8 was shown to act as a binding platform in holoenzyme HDAC complexes, suggesting a general mechanism for regulation across class I HDACs.^17–20^

**Fig. 1:**
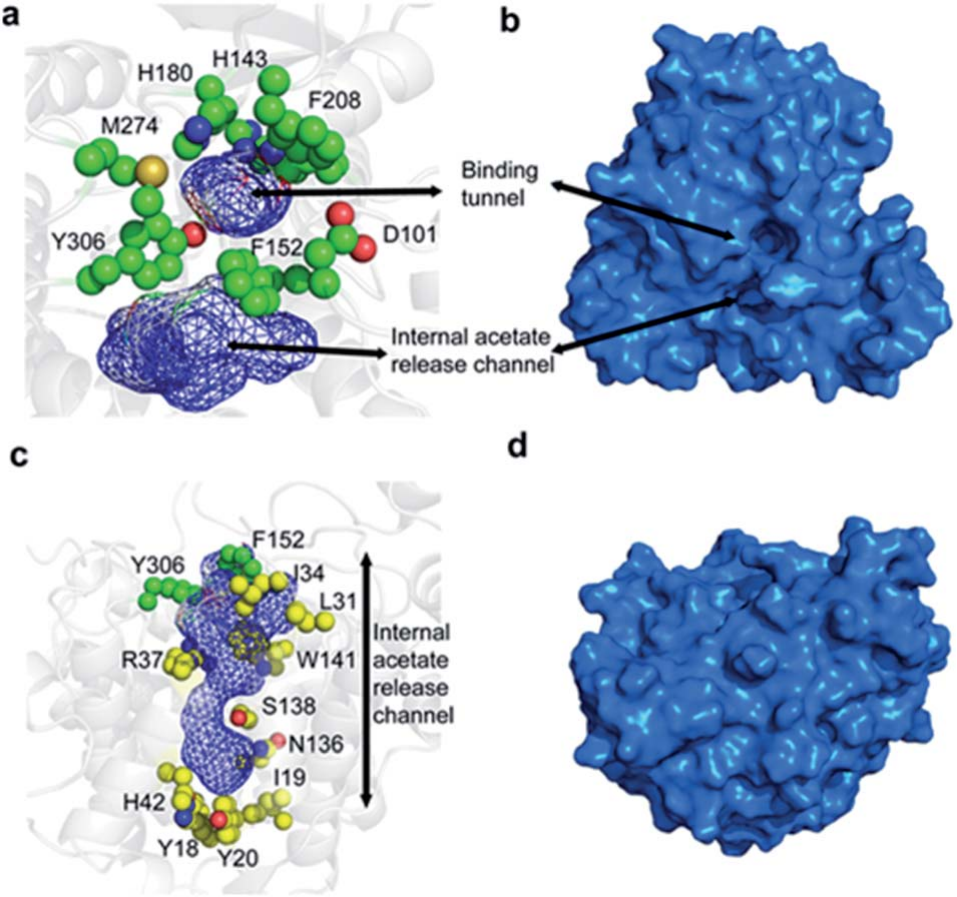
Structure of HDAC8 highlighting the binding tunnel and the proposed acetate release channel. (a) A top view of the binding tunnel and internal acetate release channel. Residues forming the wall of the binding tunnel and those present at the junction of the substrate-binding tunnel and the acetate release channel are shown in green. (b) Exterior surface representation depicting the substrate-binding tunnel in the same orientation as shown in (a). (c) Side view of the internal acetate release channel. Residues surrounding only the acetate release channel are shown in yellow. (d) Exterior surface representation of the orientation in (c).

The overall fold of HDAC8, as observed in substrate- and inhibitor-HDAC8 complexes, is comprised of an eight stranded β-sheet, which is surrounded by 11 helices and two helical turns.^21–26^ The active site is formed by seven loops, L1 to L7, connecting these secondary structure elements. The substrate-binding tunnel is formed by residues D101, H142, H143, G151, F152, H180, F208, M274, and Y306 with the active-site Zn^2+^ located at the base of the tunnel, Fig. 1a and b. These residues are conserved across the class I HDACs, except for M274, which is a leucine in the other class I family members. For class II members HDAC6 and HDAC10 a lysine and a glutamate is present, respectively, whereas a leucine is present in other class II HDAC family members at this position.^21^

It has been proposed that, in the unbound form, the active-site Zn^2+^ adopts a 5-coordinate square pyramidal geometry with D178, H180, and D267 and two water molecules.^27^ Upon substrate or inhibitor binding, one of the water molecules is displaced by the side-chain carbonyl of the substrate/inhibitor. The Zn^2+^ ion and H142 activate a water molecule for a nucleophilic attack on the carboxyl of the acetylated lysine side chain and the reaction intermediate has been proposed to be stabilised by Y306, H142, H143, and Zn^2+^. Subsequently, the hydrolysis is completed by H143 that protonates the lysine side chain.^27^

Following hydrolysis, the acetate product has been proposed to be released *via* an internal channel adjacent to the substrate-binding tunnel.^21,28^ This ‘release channel’ is lined by residues I34, F152, Y306 from the top and Y18, I19, Y20, H42, N136 from the bottom, Fig. 1c and d, with Y306, W141 and F152 located at the intersection between the substrate-binding and acetate release channel. The internal acetate release channel has been hypothesised to divide into to sub-channels with R37 and S138 present at the junction and R37 playing an important role in stabilising the negatively charged acetate for effective release. Despite the availability of many crystal structures of HDAC8 in complex with different substrates and inhibitors,^21–26^ a high-resolution structure of the unbound form of HDAC8, or any other class I HDACs, is not yet available. Also, many aspects of HDAC8 regulation and function still remain elusive,^29–32^ in particular the mechanism of product release as well as the role of the dynamic loops, L1–L7, for function and how these are connected to the active site.

In this work we performed 10 μs-long unbiased molecular dynamics simulations of wild-type HDAC8 and two mutants I19S and S39E, which are known to allosterically downregulate activity. We observe that dynamics about the *χ*_1_ dihedral angle of aromatic residues near the active site, Y306, H143, and F152, orchestrate the conformational sampling of the functional loops of HDAC8. In both mutants, I19S and S39E, the conformational sampling of the functional loops is significantly affected. In I19S-HDAC8 the dynamics of Y306 and the L6 loop is completely inhibited and a ∼20 Å allosteric path from I19 to Y306 is identified, which passes through several sites that have been identified to be essential for activity. The S39E mutant leads to a substantial stabilisation of all the aromatic side chains near the active site, which effectively traps each of the functional loops in one conformation. Overall, the results show how the active site of HDAC8 is connected to the conformational sampling of functional loops and also how mutations affect the sampled conformations and activity.

## Methods

### Experimental design

Long (10 μs) all-atom unbiased molecular dynamics simulations were used to characterise allosteric communications within the free form of the HDAC8 enzyme. The wild type HDAC8 as well as two mutants, I19S-HDAC8 and S39E-HDAC8, known to allosterically downregulate enzymatic activity were investigated to gain insight into the mechanism of downregulation. Markov state modelling was used to provide insight into the correlation between relevant motions observed.

### Structure preparation and simulation setup

To prepare the wild-type HDAC8 model the crystal structure (PDB: 1T64)^21^ was used, with the missing loops modelled in using MODELLER^33^ and the trichostatin A molecules removed. Following this, hydrogens were added and the protonation states were determined using MolProbity.^34^ The system was parameterised using the amber99SB*-ILDN forcefield with TIP3P water.^35,36^ To maintain the Zn^2+^ and K^+^ ions bound during the simulation, a harmonic potential centred around 2.25 Å, with a force constant of 3.960 J mol^−1^, was placed between the Zn^2+^ cation and H180 N^δ1^ and between the potassium distal to the active site and the carbonyl groups of V195 and T192. An *in vacuo* energy minimisation was carried out.^37^ Subsequently the system was solvated in a dodecahedral box with a volume of 458.83 nm^3^ and neutralised by adding K^+^ counter-ions followed by an additional 19 K^+^ and 19 Cl^−^ ions (70 mM). Following this, a second energy minimisation was carried out and two density equilibrations were performed. The first used a Berendsen barostat and thermostat for 100 ps.^37^ The second equilibration was carried out with the Parrinello–Rahman barostat^38^ with a Nosé–Hoover thermostat^39,40^ for 100 ps. Finally, a 2 ns NVT step was carried out to equilibrate the systems using the final run conditions with coupling to a velocity-rescaling thermostat.^41^ During all steps, the bond lengths were constrained using the LINCS algorithm^42^ and electrostatics were calculated using the PME algorithm with a 1.2 nm radius.^43^ The van der Waals interactions were determined using a standard 1.2 nm cut-off scheme. The length of each time step was 2 fs and all simulations were carried out at a temperature of 300 K. The simulations were carried out using Gromacs.^44^

The I19S and S39E simulations were prepared similar to the wild-type simulations after introducing the mutation. In order to obtain a simulation for the reverse mutation, I19Sr, the final frame from the I19S simulation was taken and S19 was mutated back to isoleucine. The waters were removed and the model was prepared as described above. Finally, the wild-type HDAC8, S39E-HDAC8 simulations were run for 10 μs, the I19S-HDAC8 simulation for 8 μs, whereas the I19Sr-HDAC8 simulation was run for 2.5 μs. The lengths of the simulations were tailored to the characteristic period of the observed conformational changes and the convergence of the sampling was also monitored through Markov state analysis. An equilibrium period of 2 μs, 1.8 μs, and 2.5 μs was used for the wild-type, S39E-HDAC8, and I19S-HDAC8 simulation, respectively. This equilibrium period was not used for any of the analyses presented and the length of the equilibrium period was determined based on the RMSD to the start structure, Fig. S1.† The loop, between K202 to V217 was not considered in the presented analysis, because convergence of this loop could not be fully justified.

### Markov state analysis

For the wild-type HDAC8 simulation, the correlation between the *χ*_1_ dynamics of H143 and Y306 was characterised using a Markov state model. Initially the *χ*_1_ angle of H143 and Y306 was calculated for every 100 ps throughout the simulation and subsequently one of the six states, {H143(g−)Y306(g−), H143(g−)Y306(t), H143(g−)Y306(g+), H143(t)Y306(g−), H143(t)Y306(t), H143(t)Y306(g+)} was assigned. Thereafter, the transition matrix, **T**, was calculated as described previously.^45^ The convergence and the appropriate lag time were assessed using the Chapman–Kolmogorov equation. First the six eigenvalues, *λ*_i_, of the transition matrix were calculated as a function of lag time, *τ*_lag_ = *n* × 100 ps and subsequently the slowest relaxation time, *t*_i_ = −*τ*_lag_/log(*λ*_i_), was calculated for different lag times, Fig. S2.† According to the Chapman–Kolmogorov equation the Markov model was assumed converged at the lag time, where the slowest relaxation time becomes independent of lag time, which was 2.8 ns. In order to calculate the transition rates the symmetrised transition matrix was used, **T̃**, and only transitions where either the side chain of H143 or Y306 flips are considered. Specifically, a least-squares minimisation of ‖**T̃** − exp(−*τ*_lag_**Γ**)‖_2_^2^, with respect to the rate constants was performed, where **Γ** is the rate matrix holding the rates.

### Calculation of Hellinger divergences

The residue-specific distributions of {*φ*,*ψ*} and *χ*_1_ in the wild-type and I19S simulation were compared using a Hellinger divergence measure. Specifically, probability distributions for {*φ*,*ψ*} and *χ*_1_, *p*(*φ*,*ψ*) and *p*(*χ*_1_) were first estimated using a von Mises distribution in Fourier space with *κ* = 120 and 120 bins over the domain *φ*,*ψ*,*χ*_1_ ∈ [−180°,180°]. Subsequently the Hellinger divergence distances, *H*, was calculated between the {*φ*,*ψ*} distribution of the wild-type and the I19S simulations, and between the *χ*_1_ distribution for the wild-type and I19S simulations by
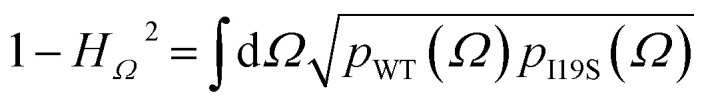
with *Ω* being either {*φ*,*ψ*} or *χ*_1_. Finally, the total Hellinger distance, *H*_T_, was calculated as*H*_T_^2^ = *H*_{*φ*,*ψ*}_^2^ + *H*_*χ*_1__^2^

A Hellinger divergence was only considered significant if it was larger than the average divergence obtained for a block-analysis, {{0 μs, 2.5 μs}, {2.5 μs, 5.0 μs}, {5.0 μs, 7.5 μs}, {7.5 μs, 10.0 μs}}, of the wild-type simulation.

All analyses was carried out using MDAnalysis,^46^ and Python 3.6 or Python 3.8 with the Numpy and Matplotlib libraries.

## Results

### Y306 and H143 each show two stable side-chain conformations

**Fig. 2:**
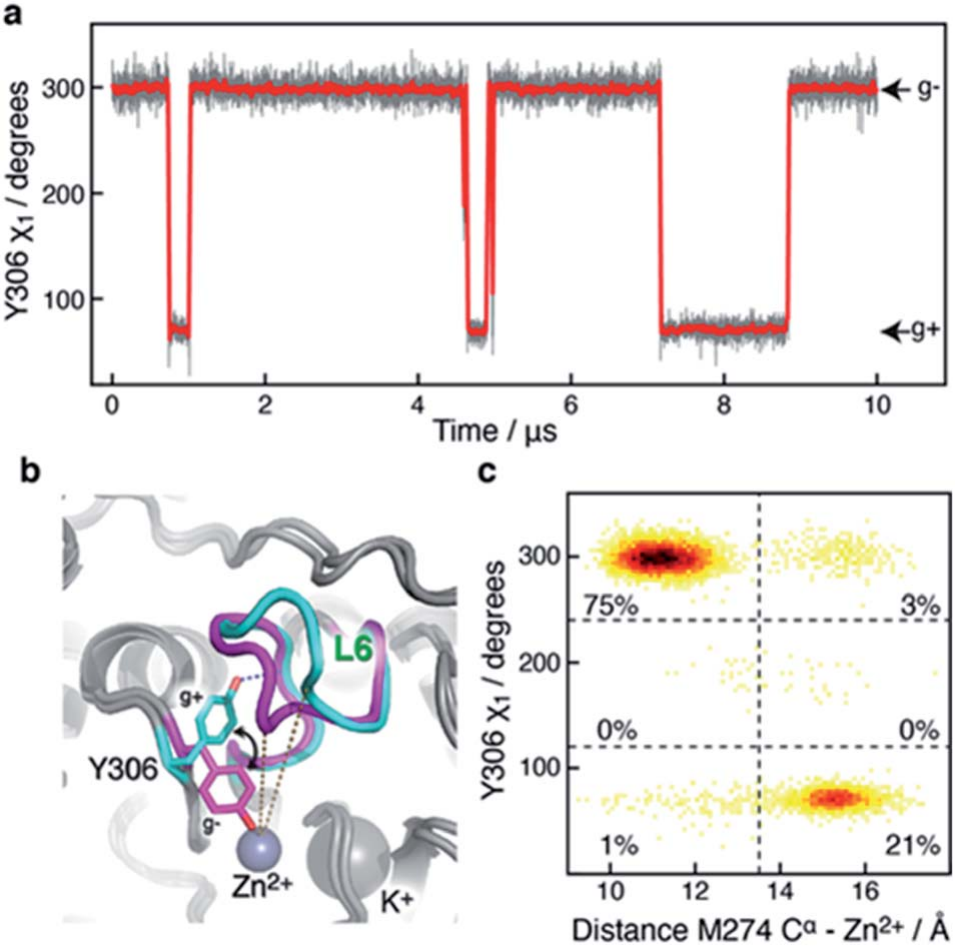
Motion of the active-site Y306 residue correlates with L6 conformation. (a) The Y306 *χ*_1_ angle as a function of simulation time (grey) and shown every nanosecond. The red line is a moving average over a window of 20 ns. Two stable and distinct conformations are observed, Y306(g−) and Y306(g+). (b) The Y306(g−) and Y306(g+) conformations are shown in magenta and cyan, respectively. The g+ conformation is stabilised by a hydrogen-bond between Y306-OH and I269 CO (blue dotted line). Moreover, the Y306 *χ*_1_ flip is associated with a large change in the L6 loop conformation. The active Zn^2+^ and the regulatory K^+^ ions are shown as grey spheres. The distance between Zn^2+^ and M274 C^α^ in two stable conformations is shown as dotted lines. (c) Two-dimensional histogram showing that the distance between M274 C^α^ within the L6 loop and Zn^2+^ correlates strongly with the *χ*_1_ dihedral angle of Y306.

A 10 μs-long atomistic molecular dynamics (MD) simulation of wild-type HDAC8 reveals reversible conformational changes of residues involved in substrate-binding and catalysis with an average lifetime of ∼2 micro-second. For Y306, which is crucial for both substrate binding and catalysis, two stable states are sampled, Fig. 2a.

**Fig. 3:**
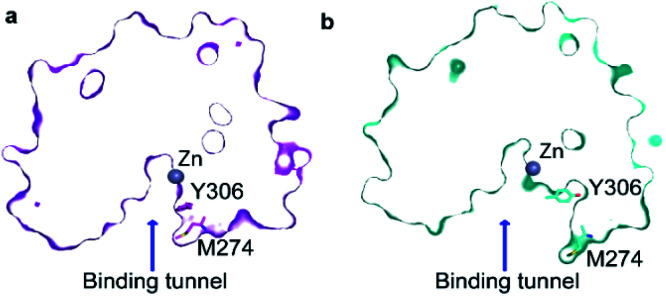
Transition from Y306(g−) to Y306(g+) leads to an open state of the active site. (a) The cut-away surface view of a representative structure of the substrate-binding tunnel in the Y306(g−) state. (b) The cut-away surface view of a representative structure of the substrate-binding tunnel and active site in the Y306(g+) state, where the active site and substrate-binding tunnel are exposed to the bulk solvent.

The major *gauche*− state (*χ*_1_ ≈ 300°; g−) is characterised by a ∼6.2 Å distance between Y306-O^ε^ and the active Zn^2+^ ion, whereas the minor *gauche*+ state (*χ*_1_ ≈ 60°; g+) is characterised by a substantial longer Y306-O^ε^ to Zn^2+^ distance of ∼11.5 Å and the tyrosine side chain pointing towards the L6 loop, Fig. 2b. The flips between the *gauche*+ and *gauche*− states of Y306 are strongly correlated with a 5-to-7 Å displacement of the L6 loop, wherein M274 is located, Fig. 2c. Loop L6 and M274 form a part of the substrate-binding tunnel and in the Y306(g+) state the tunnel is substantially wider and solvent exposed, thus showing that the *gauche*− to *gauche*+ flip of Y306 acts as a switch to open the substrate-binding tunnel, Fig. 3. Further examination of the Y306(g+) state shows that it is closely associated with a hydrogen bond between Y306-OH and I269-CO, Fig. 2b, which implies that this hydrogen bond may stabilise the Y306(g+) state. Moreover, in the Y306(g+) state a salt-bridge between D233 and R353 is fully formed, whereas in the Y306(g−) state this salt-bridge is only partly formed, Fig. S3.† The D233–R353 salt bridge, in turn, stabilises the L6 loop in the Y306(g+) state *via* additional charge–charge interactions between R353 and Cys275-CO as well as between R353 and Pro273-CO. In addition to the Y306(g−) and Y306(g+) states, there is also a very low-populated and short-lived state with the Y306 *χ*_1_ in a *trans* state (∼0.5% population), where the Y306 side chain points towards the L1 loop.

Of particular interest is that the substrate-binding tunnel is substantially widened and the active site exposed to the bulk solvent when the L6 loop is distant to the Zn^2+^, Fig. 3 and S4.† Such a state, with the exposed active site and the widened substrate-binding tunnel, will likely be able to rapidly release products. A movie for visually comparing the structure in the “closed” and “open” states, and with a transition from surface to cut-away, is shown in ESI Movie S1.†

Evidence of conformational flexibility around Y306 was previously observed in a 10 ns simulation of Y306F-HDAC8 (ref. 47) and the *gauche*+ and *trans* conformations have been observed in crystal structures of related enzymes, thereby providing evidence for the states of Y306 observed in the unbiased simulations of HDAC8 presented here. Specifically, in the crystal structure of acetylpolyamine amidohydrolase (APAH; PDB 3Q9F), *gauche*− and *trans* were observed for the corresponding residue, while in H976Y-HDAC4 the *gauche*− (PDB: 2VQV) and the *gauche*+ (PDB: 2VQO) were observed.^48,49^

**Fig. 4:**
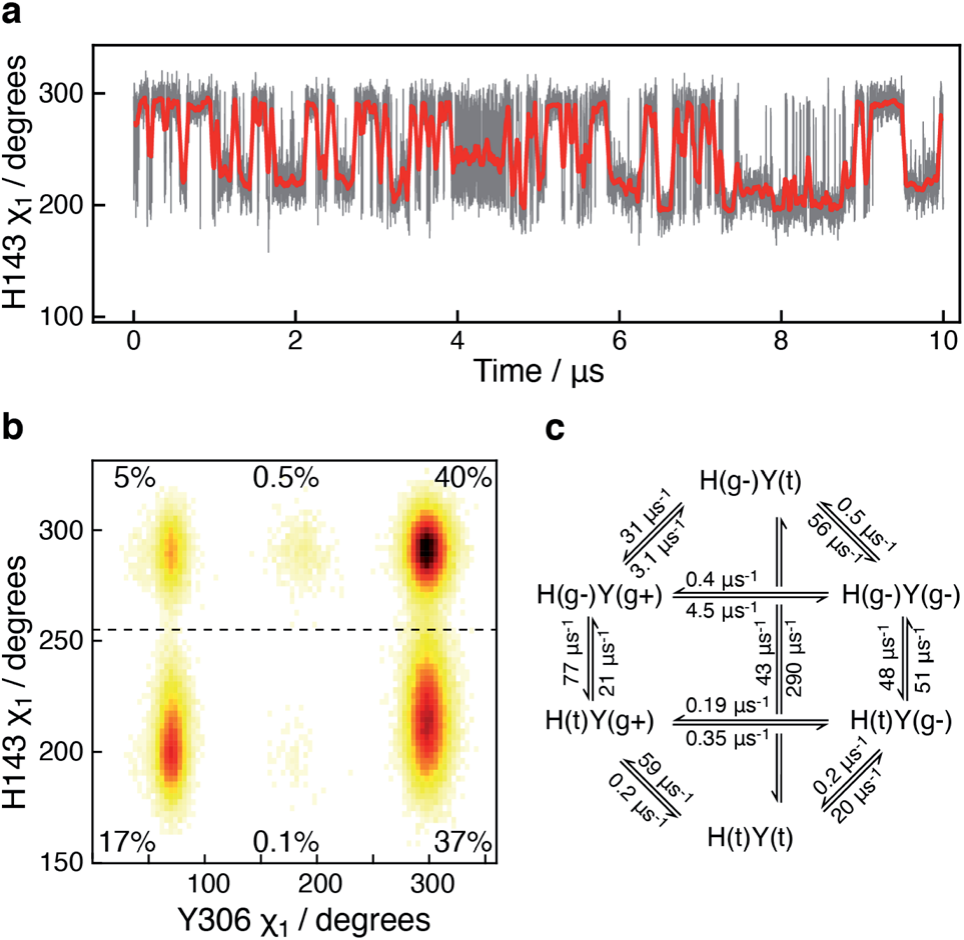
Side-chain motion of H143 correlates with Y306 sampling: (a) the H143 *χ*_1_ angle as a function of simulation time (grey) and sampled every nanosecond; the red line is a moving average over a window of 20 ns. The H143 side chain samples the *gauche*− (*χ*_1_ ≈ 300°; g−) and the *trans* (*χ*_1_ ≈ 205°; t) states each with an average life-time of 11 ns. (b) A change in the Y306 *χ*_1_ angle alters the sampling of H143 *χ*_1_ such that H143(t) is stabilised when Y306 *χ*_1_ is in a *gauche*+ conformation. (c) Markov state model derived for the H143 (H) and Y306 *χ*_1_ (Y) combined dynamics.

The active-site residue H143 is involved in the hydrolysis by forming a hydrogen-bond with the acetylated lysine N^ε^ and thus stabilising the reaction intermediate. As observed for Y306, H143 also exists in a dynamic equilibrium between two conformations that are characterised by different *χ*_1_ angles, H143(g−) with *χ*_1_ ≈ 300° and H143(t) with *χ*_1_ ≈ 205°, Fig. 4.

In all crystal structures of HDAC8, bound to a substrate or inhibitor, only the H143(g−) is present.^21–26^ When the H143 side chain flips from *gauche*− and *trans*, the entire L2 loop region (residues 86–103), as well as the L3 loop located between L2 and the active site (residues 142–154), move approximately 1 Å closer to the active site Zn^2+^, Fig. S5.† The functional L2 loop region contains D101, which is known to stabilise substrate binding.^50,51^ Moreover, a correlation is observed between the sampling of the *χ*_1_ of Y306 and H143, Fig. 4b. Specifically, when the Y306(g+) state is formed, H143 *χ*_1_ is stabilised in the *trans* conformation.

To gain further insight into how the state of Y306 influence the dynamics of H143, and *vice versa*, a Markov state model was generated from the 10 μs simulation, Fig. 4c (see Methods). The generated Markov model highlights the stabilisation of H143(t) when Y306 *χ*_1_ is in a *gauche*+ conformation, a stabilisation that appears to be mainly caused by a slower rate from H143(t) to H143(g−) and a slightly faster rate from H143(g−) to H143(t). Also, the dynamics of Y306 is essentially frozen when H143 *χ*_1_ is in the *trans* state. Taken together these observations suggest that the motions of the two functional residues, Y306 and H143, greatly influence each other and the motion of each of these aromatic side chains correlate with the conformations of the functional loops.

### The conformation of F152 steers the L1 loop

In addition to the motions of Y306 and H143 involved in binding and catalysis, similar *χ*_1_ motions were observed for F152, which forms a part of both the substrate-binding tunnel and the acetate release channel. F152 *χ*_1_ samples predominantly two states, F152(g−) and F152(t), with associated average *χ*_1_ angles of *ca.* 300° and *ca.* 200°, respectively.

**Fig. 5:**
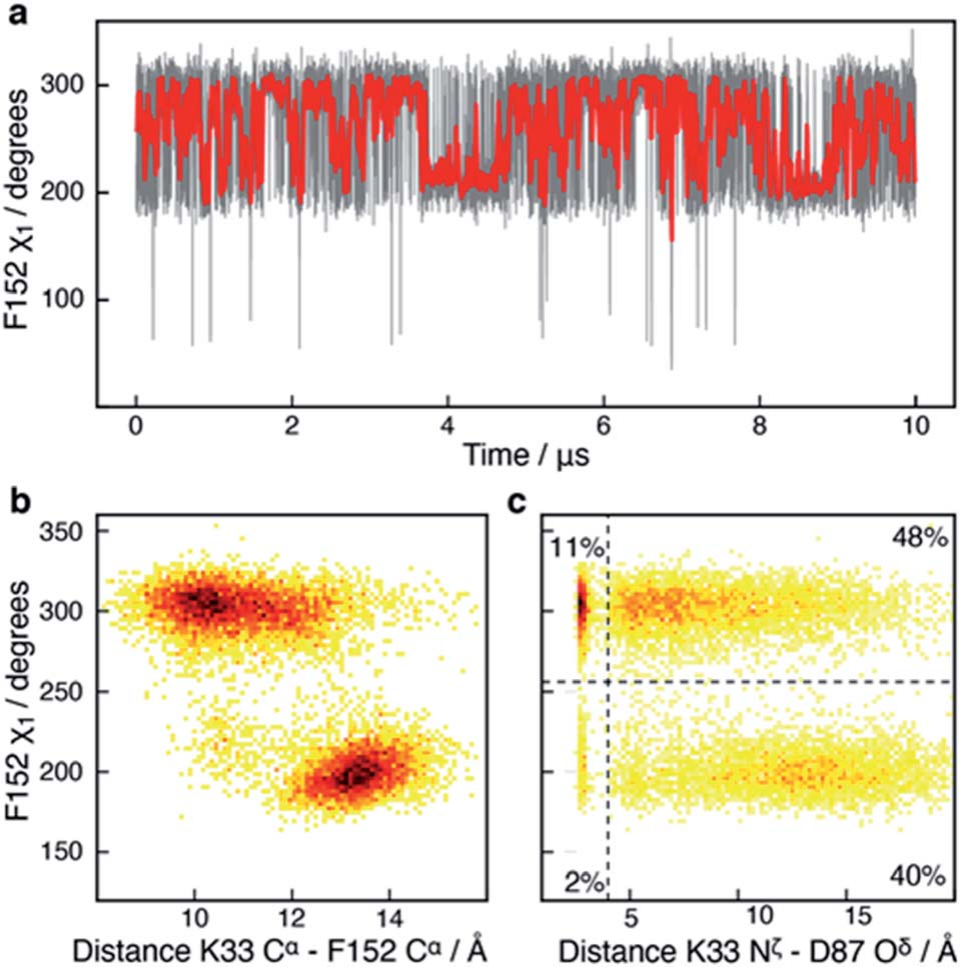
Side-chain motion of F152 correlates with L1 sampling: (a) the F152 *χ*_1_ angle as a function of simulation time (grey) and sampled every nanosecond; the red line is a moving average over a window of 20 ns. The F152 side chain samples the *gauche*− (*χ*_1_ ≈ 300°; g−) and the *trans* (*χ*_1_ ≈ 200°; t) states each with an average life-time of about 1 ns. (b) Two-dimensional histogram showing that the *χ*_1_ dihedral angle of F152 correlates strongly with the distance between K33 C^α^ within the L1 loop and F152 C^α^; a distance which has previously been used as a measure of the L1 conformation.^17^ (c) Two-dimensional histogram showing that the distance between the side chains of K33 in L1 and D87 in L2 as well as ability to form a salt-bridge between the two side chains, correlate with the *χ*_1_ of F152. In the F152(t) state, a salt-bridge between L1 and L2 is unlikely (5%), whereas in the F152(g+) state there is a 22% chance of a salt-bridge being formed.

The *χ*_1_ motion of F152 is substantially faster than those of H143 and Y306, with F152(g−) and F152(t) having an average life-time of 1.4 ns and 1.1 ns, respectively, during the 10 μs simulation, Fig. 5. Thus, there is a slight preference for the F152(g−) state (58%) over the F152(t) state (42%). Moreover, the F152(t) state is stabilised twice, for approximately 1 μs each time, Fig. 5a. The F152(g+) state is only present at 0.3%.

The F152 residue is found in the *gauche*− conformation in all human HDAC8 crystal structures solved so far, however, in the HDAC8 homolog from *Schistosoma mansoni* the corresponding phenylalanine residue, F151, is observed in a *trans* state,^52^ see Fig. S6,† thereby providing evidence that the substantial amount of *trans* conformation observed in the unbiased simulations is realistic for HDAC8 without a substrate or an inhibitor bound.

The correlation between the conformation of the L1 loop and the *χ*_1_ angle of F152 is striking, Fig. 5b. When F152 is in the *trans* state, the L1 loop moves approximately 3 Å away from the substrate-binding tunnel leading to an open state. In line with this movement is that the salt-bridge between K33 in L1 and D87 in L2, previously related to function,^50^ predominantly forms in the F152(g−) state. These findings agree with the crystal structure of the HDAC8 homolog from *Schistosoma mansoni*, where the corresponding phenylalanine is in *trans*, and the L1 loop is *ca.* 1 Å further away from the active site compared to human HDAC8.

Despite the motion about the F152 *χ*_1_ angle being fast, the conformation of other functional sites is correlated with the state of the F152 side chain. One example is R37, Fig. S7,† which has previously been suggested to play a central role for the release of acetate. In all HDAC8-inhibitor or substrate complexes, the guanidinium group of R37 forms hydrogen bonds with the backbone carboxyl oxygen atom of two conserved glycine residues (G303 and G305) in the glycine rich loop adjacent to Y306.^21–26^ These hydrogen bonds are formed throughout the HDAC8 simulation, however motion around the R37 *χ*_1_ angle was observed to be affected by the F152 *χ*_1_ sampling, Fig. S7.† Despite the ∼10 Å distance between F152 and R37, both residues are in proximity of the L1 loop, suggesting that the motions of F152 and R37 could be correlated with each other *via* the L1 loop.

In crystal structures of HDAC8–ligand complexes, both Y306 and F152 are present at the junction of the substrate-binding tunnel and the acetate release channel, and changes in these two residues are important for the opening of the acetate release channel.^21,28^ Despite this, only a weak correlation is observed between Y306 *χ*_1_ and F152 *χ*_1_ as well as between Y306 and R37, Fig. S8.† Among all the residues in the vicinity of the substrate-binding tunnel, the active site, and the product release channel, *χ*_1_ flips are effectively only observed for the four residues described above, *i.e.*, R37, H143, F152, and Y306. For all the other residues near the active site one *χ*_1_ conformation dominates, see Fig. S9.†

### The S39E mutation alters the sampling of both L1 and L6

The side-chain *χ*_1_ flips of Y306, H143, and F152 described above report on the catalytic cycle of HDAC8 and the motions relate to substrate binding and product release. It is therefore of interest to investigate how these side-chain *χ*_1_ motions are potentially altered in mutants of HDAC8 known to be substantially downregulated. In addition to the 10 μs simulation of wild-type HDAC8, a 10 μs simulation was also carried out for the phosphorylation mimicking mutant S39E of HDAC8. Phosphorylation of HDAC8 at S39 as well as the S39E mutation lead to a downregulation^21,53^ of HDAC8 enzymatic activity and since S39 is located near the L1 loop it is of interest to investigate the mechanism by which this mutation alters the dynamic sampling of the functional loops.

**Fig. 6:**
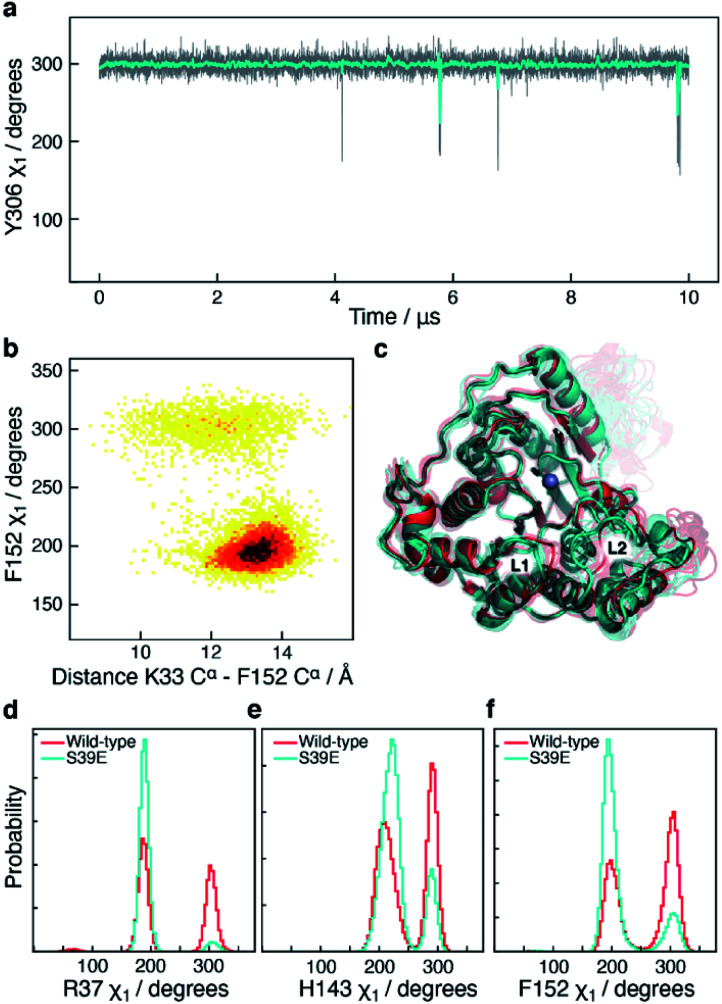
Simulation of S39E-HDAC8 phosphorylation mimicking mutant. (a) The Y306 *χ*_1_ angle as a function of simulation time (grey) and sampled every nanosecond during the S39E-HDAC8 simulation. The cyan line is a moving average over a window of 20 ns. Only the Y306(g−) and the Y306(t) states are sampled, with probabilities of 99.6% and 0.4%, respectively. (b) Two-dimensional histogram depicting the correlation between the distance between K33 C^α^ within the L1 loop and F152 C^α^, showing that the L1 loop is consistently distant to the substrate-binding tunnel and active site. (c) Average structure of the S39E-HDAC8 simulation in the most stable conformation, R37(t), H143(t), F152(t), Y306(g−), (cyan), along with 10 representative frames (semi-transparent cyan). Average of wild-type HDAC8 simulation in the R37(g+), H143(g+), F152(g+), Y306(g−) conformation (red), along with 10 representative frames (semi-transparent red). (d)–(f) Histograms show a substantial higher preference for R37, H143, and F152 *χ*_1_ to sample the *trans* conformation in the S39E simulation.

In the S39E-HDAC8 simulation, the dynamic sampling of Y306 *χ*_1_ and the L6 loop is inhibited and effectively only sample the *gauche*− state and the proximal conformation, respectively, Fig. 6a. Moreover, the F152 *χ*_1_ angle is highly stabilised in the *trans* state throughout the S39E simulations, which in turn leads to L1 conformations consistently distant to the active site and a very low probability of forming the K33 N^ζ^–D87 O^δ^ salt-bridge, Fig. 6b–f. A Markov state model similar to Fig. 4c cannot be derived from the S39E-HDAC8 simulation, because the Y306(g+) state is not populated and the Y306(t) state is only present in a few frames. With regards to the kinetics observed for H143 *χ*_1_, the H143(g−) and H143(t) states have average lifetimes of 0.023 μs and 0.048 μs, respectively, during the S39E-HDAC8 simulation compared to 0.020 μs and 0.024 μs in the simulation of wild-type HDAC8.

### The I19S CdLS mutation prevents an opening of the L6 loop

**Fig. 7:**
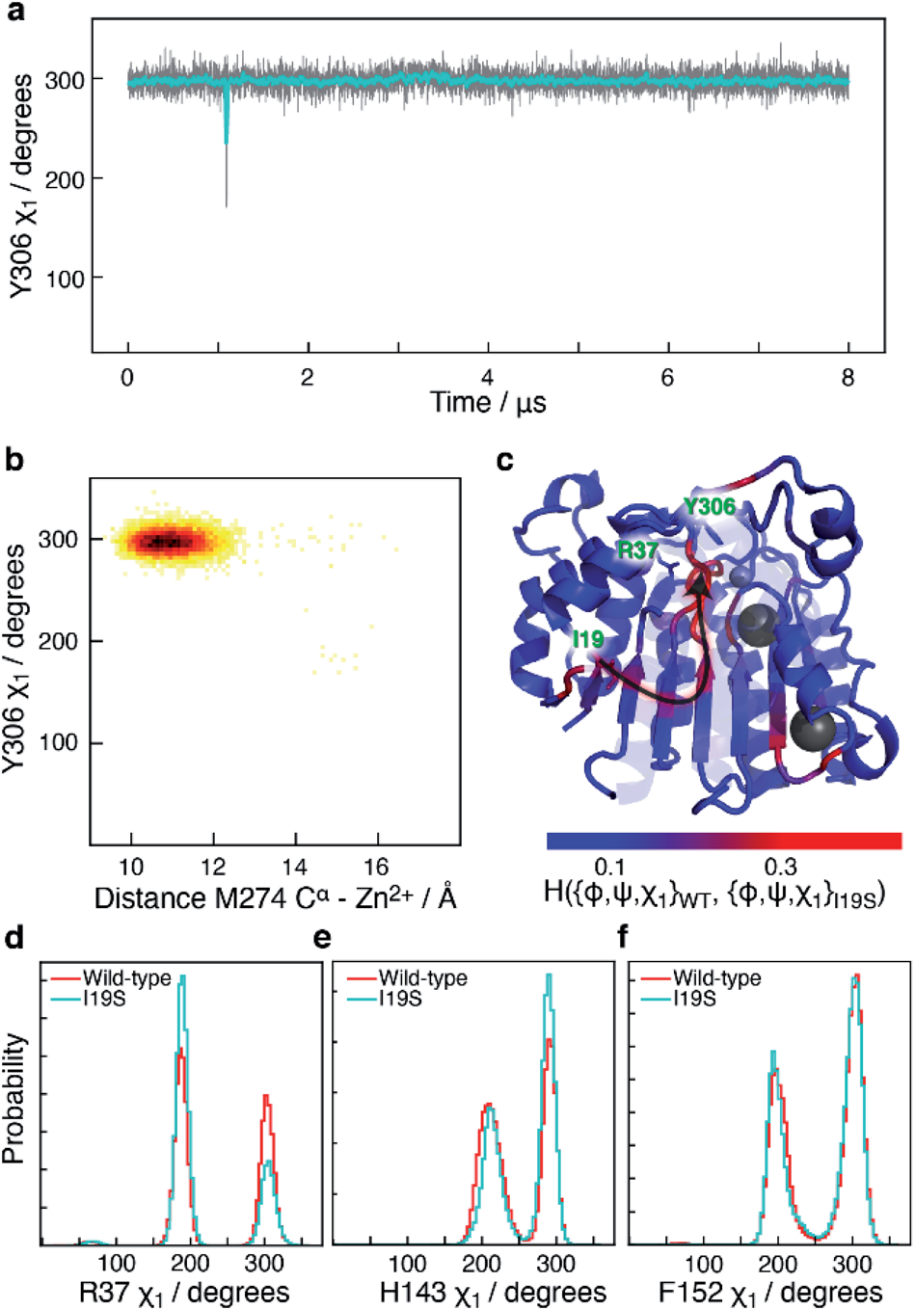
Simulation of I19S-HDAC8 and comparison with wild-type. (a) The Y306 *χ*_1_ angle as a function of simulation time (grey) and sampled every nanosecond in the I19S-HDAC8 simulation. The cyan line is a moving average over a window of 20 ns. Only the Y306(g−) and the Y306(t) states are sampled, with probabilities of 99.9% and 0.1%, respectively. (b) Two-dimensional histogram showing the correlation between the distance between M274 C^α^ within the L6 loop and Zn^2+^, and Y306 *χ*_1_. The L6 loop only samples the conformation near the active site. (c) Residue-specific Hellinger distances between the {*φ*,*ψ*,*χ*_1_} distribution in the I19S and wild-type simulations shown on the structure of HDAC8 (PDB: 2V5W).^22^ A clear path between the site of mutation, I19, and Y306 is seen. (d) A slightly higher preference for a R37 *χ*_1_*trans* conformation in the I19S simulation. (e) and (f) Very similar probability distributions in the I19S- and wild-type simulation for the H143 and F152 *χ*_1_.

Another mutant, which has been reported in CdLS patients^12^ and which leads to a substantial downregulation of HDAC8 is I19S. I19S-HDAC8 has an activity of <10% compared to wild-type HDAC8. The I19 residue is located at the base of the acetate release channel, Fig. 1, and about ∼21 Å from the active site. An 8 μs MD simulation was carried out for the I19S mutant of HDAC8 (I19S-HDAC8). The first striking difference between the wild-type HDAC8 and I19S-HDAC8 simulation is that Y306, and consequently the L6 loop, are completely rigidified in the I19S-HDAC8 simulation, Fig. 7a and b. From the discussion above, this means that I19S-HDAC8 does not sample a state with an open conformation of the L6 loop, which in turn relates to product release. To rule out that the I19S-HDAC8 simulation had got trapped in a local minimum with Y306 in a *gauche*− state, the last frame of the I19S-HDAC8 simulation was taken out and residue 19 mutated back to isoleucine, thereby recovering the wild-type sequence. A new simulation was then initiated starting from this final frame of the I19S-HDAC8 simulation, but now with residue 19 being isoleucine. After about 1 μs of simulation time, of the back-mutated structure, Y306 starts to sample the *gauche*+ state, thus substantiating that the inhibition of the Y306 and L6 movements is caused by the I19S mutation, Fig. S10.† As for the S39E-HDAC8 simulations, a Markov state model similar to Fig. 4c cannot be derived from the I19S-HDAC8 simulation. With regards to the kinetics observed for H143 *χ*_1_, the H143(g−) and H143(t) states have average lifetimes of 0.013 μs and 0.009 μs, respectively, during the I19S-HDAC8 simulation, which are significantly shorter compared to 0.020 μs and 0.024 μs observed in the simulation of wild-type HDAC8.

I19 and Y306 are more than 20 Å apart, both in the simulations and in all published structures of HDAC8. A central question is therefore how the I19S mutation leads to an inhibition of the *χ*_1_ motions of Y306 as well as the motions of the L6 loop. To gain insight into the differences between the I19S and wild-type simulations, Hellinger distances were calculated between the backbone {*φ*,*ψ*} and side-chain *χ*_1_ distributions in the I19S and wild-type simulations, for each residue. A block analysis was also performed on the wild-type simulation and a Hellinger distance (I19S to wild-type) was only considered significant if it was significantly larger than what was obtained in the block analysis of the wild-type simulations (see Methods). When the significant Hellinger distances (I19S *versus* wild-type) are shown on the structure of HDAC8, Fig. 7c, a clear path is observed between residue 19 and Y306. It is important to note here that this path appears naturally and has not in any way been imposed during the calculation of the Hellinger distances. The observed path goes through the central β-sheet of HDAC8, including residues I135 and L299, from where the path runs *via* the glycine loop, G302–G305 to Y306. The glycine loop, G302–G305, has previously been implicated in function and mutating any of these glycine residues to alanine leads to a small change in the loop conformation in the resulting crystal structures in bound form, but causes a substantial effect on HDAC8 activity, with only about 0.3–3% of enzymatic activity retained.^47^ Thus, not only do the Hellinger distances provide insight into how the effect of the I19S mutation is transmitted though the structure of HDAC8, but also highlight areas where mutations and regulator binding could alter HDAC8 activity due to a change in the L6 dynamics.

Arginine R37 forms interactions with the glycine loop, G302–G305. Although there is no significant change in the hydrogen-bonding between the guanidinium group of R37 and the glycine loop in the I19S-HDAC8 and wild-type HDAC8 simulations, the distribution of R37 *χ*_1_ changes slightly between the two simulations, Fig. 7d, consistent with the allosteric path going through the glycine loop. In contrast, only limited changes are observed for the distribution of the side chain *χ*_1_ of H143 and F152 and thus of the L1 and L2 loops, Fig. 7e and f. The motion about the F152 *χ*_1_ dihedral angle is slightly faster in the I19S-simulation, where the F152(g−) has a lifetime of 1.0 ns compared to 1.4 ns in the wild-type simulation.

## Discussions

Several hypotheses have previously been suggested for the mechanism of deacetylation and downregulation of HDAC8 activity for different CdLS mutants on the basis of the structure of wild-type HDAC8 and mutants bound to various inhibitors/substrates. However, the mechanism of product release as well as the role of the dynamic loops in catalysis and how the dynamics of these are connected to key active-site residues remain elusive. Also, the effects of the various CdLS mutations and phosphorylation on the dynamics of these loops have remained largely unknown.

A substantial change in the dynamics of the L1 and L6 loops was observed upon investigation of a CdLS down-regulating mutant, I19S, as well as a phosphorylation mimicking mutation, S39E, of HDAC8. In the S39E-HDAC8 simulation all the aromatic side chains near the active site were highly stabilised in one conformation leading to a consistent open form of the L1 loop and a closed conformation of the L6 loop. Recently, Michaelis–Menten parameters, *k*_cat_ and *K*_M_, were reported for the S39E mutant of HDAC8, where changes in both *k*_cat_ (∼3 fold decrease) and *K*_M_ (∼2 fold increase) were observed.^53^ A change in *K*_M_ upon mutation typically reflects a change in the substrate-binding affinity, while a change in *k*_cat_ reflects a change in the rate of hydrolysis and/or a change in the rate of product release. These experimental data therefore suggests that mutating S39 to glutamate affects both substrate-binding as well as hydrolysis and/or product release. In the MD simulations presented above, changes in sites related to both substrate-binding (L1; F152), hydrolysis (H143), and product release (L6; Y306, M274) were observed, in line with the experimental findings.

In the simulation of I19S-HDAC8, the backbone conformation of G302–G305 changed slightly and the L6 loop as well as Y306 were trapped in a closed conformation. In available experimental enzyme kinetics data, mutation of Y306 to phenylalanine resulted in a two-fold decrease in the *K*_M_ and a 290-fold decrease in the catalytic constant, *k*_cat_.^54^ Similarly, upon mutation of glycine residues adjacent to Y306 to alanine, G304A and G305A, a ∼300 and a ∼38 fold decrease in *k*_cat_ was observed, respectively, with only a 2 fold and a 1.5 fold increase in *K*_M_.^47^ Therefore, the predominant role of Y306, whose conformational sampling is affected by the adjacent glycine residues, is in stabilising the enzymatic transition state and/or in the release of products, *k*_cat_, as opposed to substrate-binding, *K*_M_.^54^ In the crystal structure of the G304A and G305A mutants, in complex with an inhibitor, only a minor change in the position of Y306 is observed relative to wild-type structures. Importantly, in these structures there are no significant change in the distance between Y306-O^ε^ and the carboxyl of the inhibitor: 2.4 Å in wild-type HDAC8 and 2.6 Å in G304A-HDAC8 and G305A-HDAC8. This strongly suggests that these mutations have limited effect on transition-state stabilisation. Hence, the glycine-to-alanine mutations likely change *k*_cat_ by impeding the release of product, which in turn happens *via* a change in the conformational sampling of Y306. Based on comparison of these experimental biochemical data with the results of the molecular dynamic simulations, it is reasonable to hypothesise that the downregulation caused by the I19S mutation is due to an inhibition of the L6 dynamics and a slight change in the sampling of R37, which results in the inability of I19S-HDAC8 to release the products.

During the wild-type HDAC8 simulation the Y306(g−) state, which has the substrate-binding tunnel formed, is the most stable state with an average lifetime of 1.3 μs. Within the Y306(g−) state, R37, H143 and F152 are in rapid dynamic equilibrium between their *gauche*− and *trans* states and we therefore propose that the Y306(g−) state is a substrate-binding-competent state. In all crystal structures of HDAC8 bound to substrate or inhibitor, R37, H143, F152 and Y306 are all found in the *gauche*− state. A comparison of the HDAC8 frames with the side chain *χ*_1_ of R37, H143, F152 and Y306 in the *gauche*− state with the crystal structures shows some differences for residues forming the active site, the substrate-binding tunnel and the release tunnel. This includes the orientation of W141 and Y306, Fig. S11.†

**Fig. 8:**
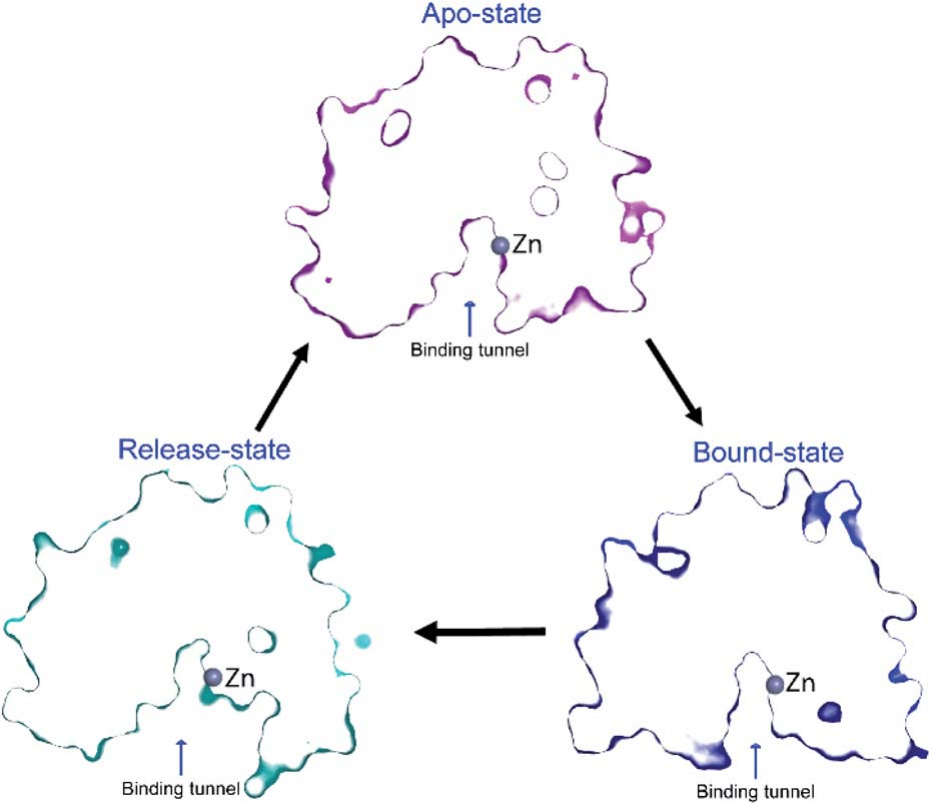
Change in structure of binding tunnel of HDAC8 during catalysis. The cut-away surface views of representative structures of HDAC8 in the apo-state: {R37(g−), F152(g−), Y306(g−)} (top), release-state: {R37(t), F152(t), Y306(g+)} (bottom left), and bound-state: crystal structure 2V5W^22^ (bottom right).

However, it is very likely that these side chains move slightly in the presence of substrate, to stabilise the substrate for catalysis, including forming a hydrogen-bond between Y306 and the substrate carboxyl oxygen. The Y306(g+) state can be considered as a product-release state, since the substrate-binding tunnel is widely exposed (see ESI Movie S2†). This agrees with the experimental enzyme kinetics data available for Y306F-HDAC8, where only the catalytic rate, *k*_cat_, is substantially affected whereas binding of substrate is only slightly affected. A concomitant movement of R37 and F152 *χ*_1_ to *trans* in the Y306(g+) state facilitates the release of acetate. The structure of the binding tunnel in the three states: apo-state, bound-state, and release-state are shown in Fig. 8.

## Conclusions

We obtained several 10 μs-long molecular dynamics simulations and analysed them to characterise the link between functional loops and the active site of HDAC8 as well as the effect of clinically relevant mutations on them. We observed that the conformation of aromatic side chains near the active site of HDAC8 orchestrate the sampling of the functional loops L1, L2 and L6. Specifically, side-chain movements of Y306 correlate strongly with an opening and closing of the L6 loop and side-chain *χ*_1_ flips of F152 strongly correlate with an opening and closing of the L1 loop. The opening and closing of the functional loops relate to the catalytic cycle of HDAC8, including binding of substrate and release of products. Overall, the aromatic side-chain *χ*_1_ movements provide the HDAC8 enzyme with a means to connect the active site with the functional loops. The presented link between functional loops and aromatic residues in the vicinity of the active site of HDAC8 provides a conceptual platform by means of which a mechanism for the regulation of HDAC8 can be derived and the mechanism of mutants from genetic disorders such as CdLS can be rationalised.

## Data availability

The trajectories associated with the research presented here are available from the corresponding author upon reasonable request.

## Author contributions

V. K. S., L. S., and D. F. H. designed the research, V. K. S. and L. S. performed molecular dynamics simulations, all authors analysed the data and molecular dynamics trajectories, and V. K. S., L. S. and D. F. H. wrote the paper. All authors discussed the results, commented on the paper, and have given approval to the final version of the manuscript. These authors contributed equally to this work: Vaibhav Kumar Shukla, Lucas Siemons.

## Conflicts of interest

The authors declare that they have no conflicts of interest with the content of this article.

## Supplementary Material

SC-012-D1SC01929E-s001

SC-012-D1SC01929E-s002

SC-012-D1SC01929E-s003
