# Supplementary material for: Aromatic side-chain flips orchestrate the conformational sampling of functional loops in human histone deacetylase 8
Source: Chem Sci. 2021 May 27;12(27):9318–27. doi: 10.1039/d1sc01929e (PMC8278956; doi:10.1039/d1sc01929e)
Supplement: SC-012-D1SC01929E-s003 [file SC-012-D1SC01929E-s003.pdf]

Electronic Supplementary Material (ESI) for Chemical Science.
This journal is © The Royal Society of Chemistry 2021

Supplementary Materials for

Aromatic side-chain flips orchestrate the conformational sampling of functional loops in Human Histone Deacetylase 8

Vaibhav Kumar Shukla, Lucas Siemons, Francesco L. Gervasio, D. Flemming Hansen*

*Corresponding author. Email: d.hansen@ucl.ac.uk

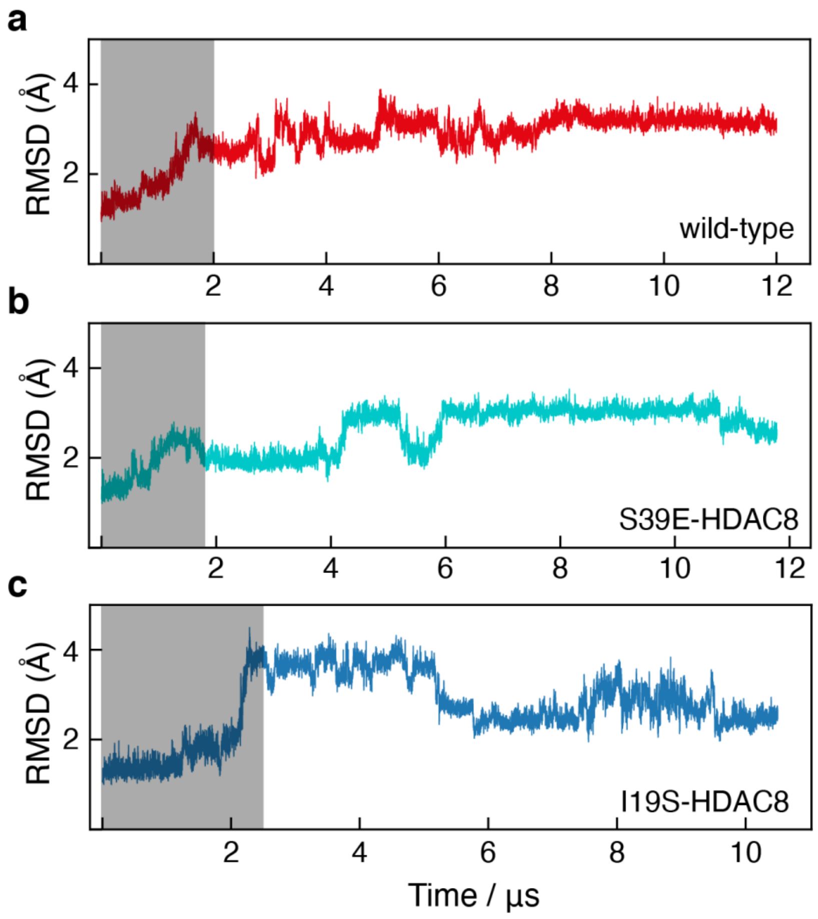

Fig. S1. Root-mean-square-deviation (r.m.s.d.) to starting structure for the three unbiased simulations. (a) RMSD for the wild-type simulation. A 2 µs equilibrium time was chosen and 10 µs production simulation (b) RMSD for the S39E-HDAC8 simulation. A 1.8 µs equilibrium time was chosen leading to 10 µs production simulation. (c) RMSD for the I19S-HDAC8 simulation. A 2.5 µs equilibrium time was chosen followed by 8 µs production simulation.

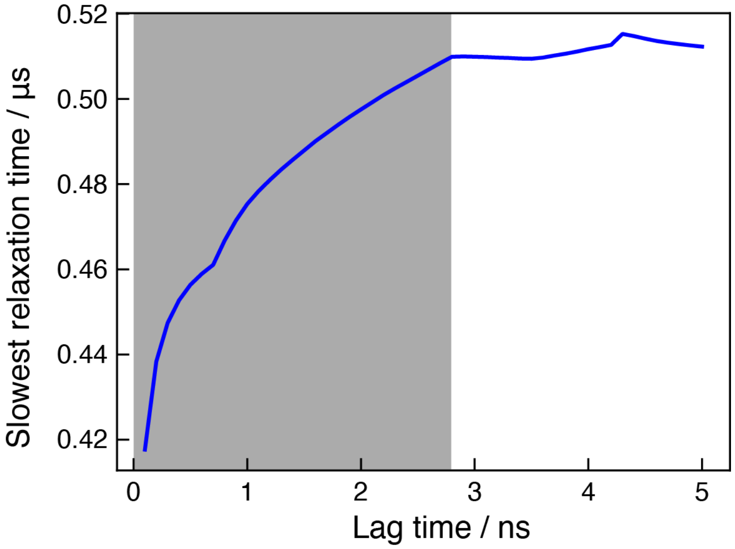

Fig. S2. Assessment of the convergence of the Markov state model. Shown is the slowest relaxation time of the transition matrix as a function of the lag time (see main text). The Markov state model is considered converged, when the slowest relaxation time becomes independent of lag time, that is 2.8 ns.

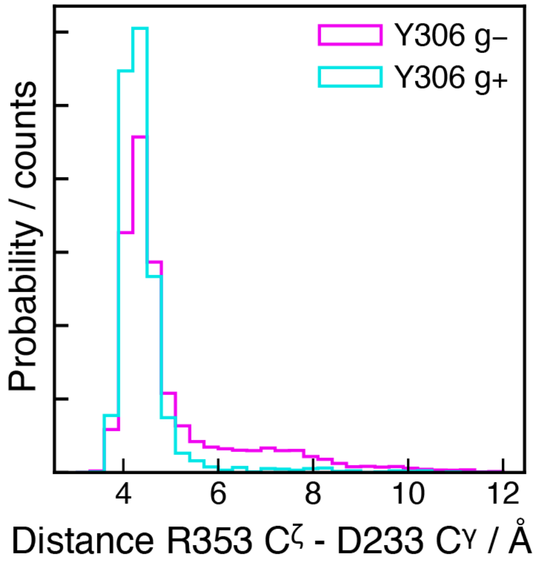

Fig. S3. Salt-bridge formation between R353 and D233. Probability distributions of salt bridge formation between R353 and D233 depending on the side-chain conformation of Y306. The salt bridge is fully formed, when the Y306 side-chain χ1 angle is in the gauche+ (g+) conformation.

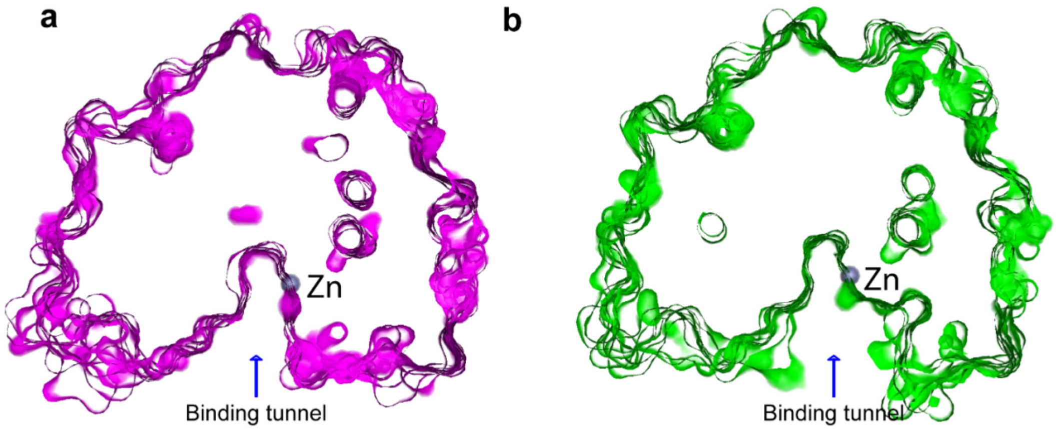

Fig. S4. Transition from Y306(g-) to Y306(g+) leads to an open state of the active site. (a) The cut-away surface views of ten random and representative structures of the substrate-binding tunnel in the Y306(g-) state. (b) The cut-away surface views of ten random and representative structures of the substrate-binding tunnel and active site in the Y306(g+) state, where the active site and substrate-binding tunnel are exposed to the bulk solvent.

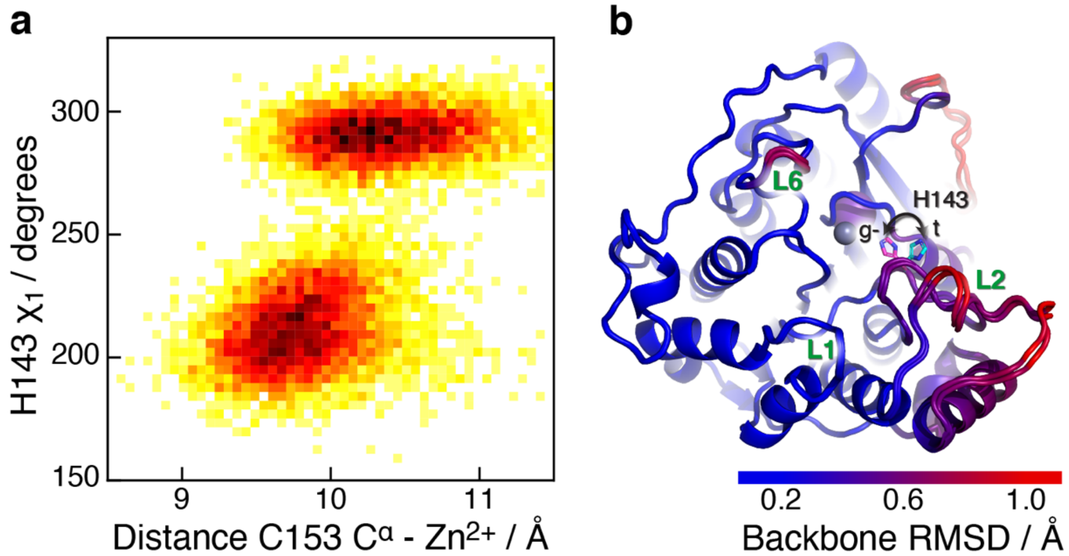

Fig. S5. Correlation between H143 χ1 and the conformation of loop L2. (a) The distance between C153 Cα within the L3 loop region and Zn^{2+} correlates with the χ_1 dihedral angle of H143. (b) The RMSD between the average H143(g-) and H143(t) conformations is shown. The χ_1 flip of H143 from *trans* to *gauche*- leads to the L2 region moving out and away from the active site by approximately 1 Å. The H143(g-) and H143(t) conformations are shown in magenta and cyan, respectively and the functional loops, L1, L2, and L6 labelled.

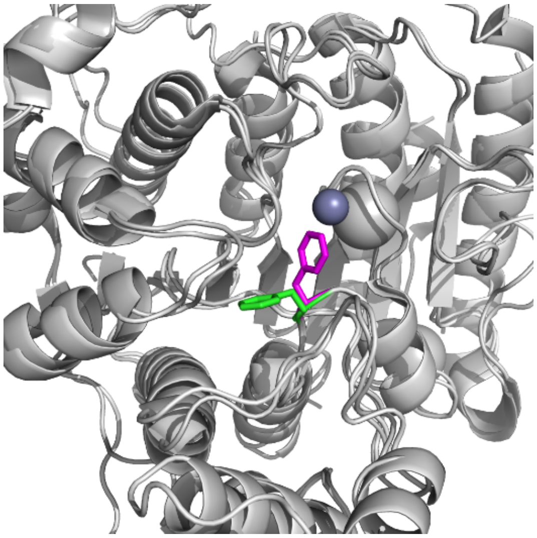

Fig. S6. Side-chain conformation of F152. Shown is residue F152 in human HDAC8 with χ_1 in *gauche-* (magenta; PDB: 1T69), and the corresponding F151 residue in the HDAC8 homologue from *Schistosoma mansoni* with χ_1 in *trans* (green; PDB: 6GXA).

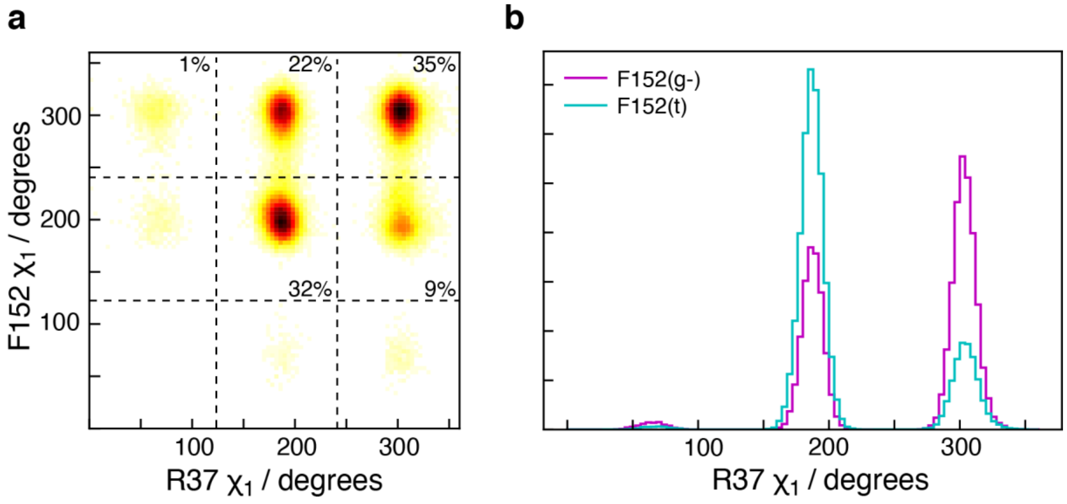

Fig. S7. Correlation between side-chain motions of F152 and R37. (a) Distribution of R37 and F152 χ_1 dihedral angles. The χ_1 dihedral angle of F152 affects the sampling of R37; a residue which has been implicated in the release of the acetate product. (b) Histogram showing that the sampling of R37 χ_1 depends on the χ_1 side-chain conformation of F152. When F152 χ_1 is in a trans conformation the R37 χ_1 angle is stabilised in the *trans* conformation.

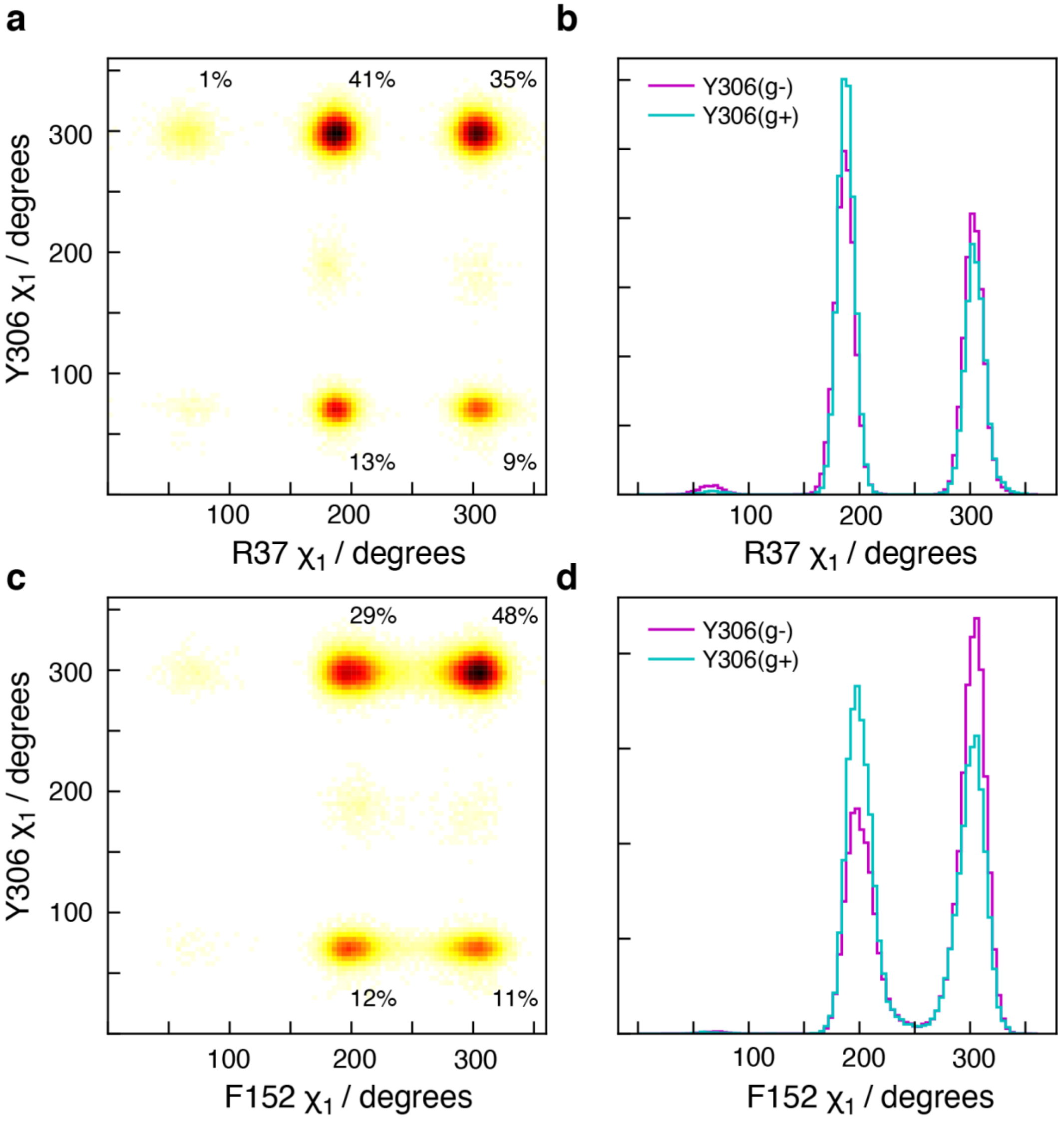

Fig. S8. Correlation between side-chain motions of Y306 and F152 and R37. (a) Two-dimensional histogram showing the combined distribution of R37 and Y306 χ_1 dihedral angles. The χ_1 dihedral angle of Y306 shows minimal correlation with the sampling of R37. (b) Change in the sampling of R37 χ_1, depending on the side-chain conformation of Y306. (c) Two-dimensional histogram showing that the χ_1 dihedral angle of F152 is weakly correlated with the sampling Y306 χ_1. (d) Change in the sampling of F152 χ_1, depending on the side-chain conformation of Y306.

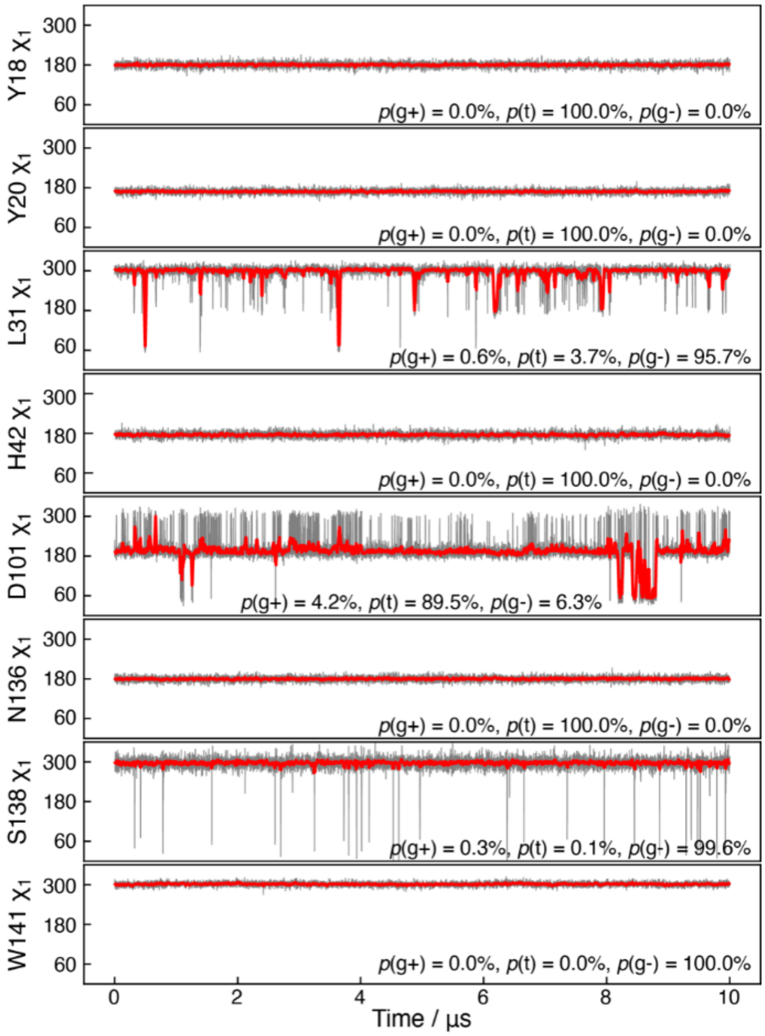

Fig. S9. Dihedral χ_1 angle for other residues. The χ_1 angle for residues Y18, Y20, L31, H42, D101, N136, S138, and W141, which are all located at the binding tunnel or the internal acetate release channel, as a function of simulation time (grey) and sampled every nanosecond. The red lines are moving averages over a window of 20 ns.

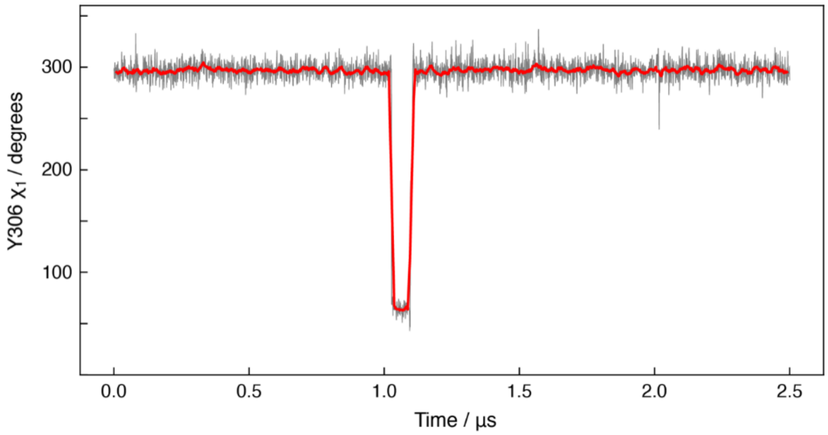

Fig. S10. Sampling of Y306 in the back-mutated S → I mutation of the I19S-HDAC8 simulation. The Y306 χ_1 angle as a function of simulation time (grey) and shown every nanosecond. The red line is a moving average over a window of 20 ns. As was seen in the initial simulation of wild-type HDAC8, two stable distinct conformation are observed, Y306(g-) and Y306(g+).

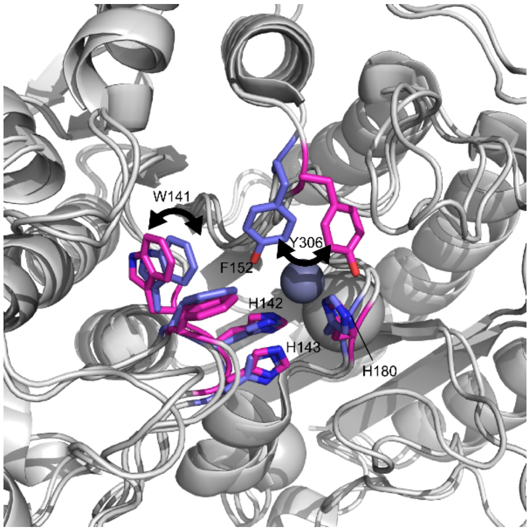

Fig. S11. Change in side-chain orientation of various residues in the bound- to apo-state transition. Side chains of the residues in the “apo-state” and in the “bound-state” are shown in magenta and blue colour, respectively.
